# Super-resolved fluorescence imaging of peripheral nerve

**DOI:** 10.1038/s41598-022-16769-0

**Published:** 2022-07-21

**Authors:** Iván Coto Hernández, Suresh Mohan, Steven Minderler, Nate Jowett

**Affiliations:** Surgical Photonics and Engineering Laboratory, Massachusetts Eye and Ear, Harvard Medical School, 243 Charles St, Boston, MA 02114 USA

**Keywords:** Anatomy, Diseases, Neurology, Confocal microscopy, Scanning electron microscopy, Super-resolution microscopy

## Abstract

Traditional histopathologic evaluation of peripheral nerve employs brightfield microscopy with diffraction limited resolution of ~ 250 nm. Though electron microscopy yields nanoscale resolution of the nervous system, sample preparation is costly and the technique is incompatible with living samples. Super-resolution microscopy (SRM) comprises a set of imaging techniques that permit nanoscale resolution of fluorescent objects using visible light. The advent of SRM has transformed biomedical science in establishing non-toxic means for investigation of nanoscale cellular structures. Herein, sciatic nerve sections from GFP-variant expressing mice, and regenerating human nerve from cross-facial autografts labelled with a myelin-specific fluorescent dye were imaged by super-resolution radial fluctuation microscopy, stimulated emission depletion microscopy, and structured illumination microscopy. Super-resolution imaging of axial cryosections of murine sciatic nerves yielded robust visualization myelinated and unmyelinated axons. Super-resolution imaging of axial cryosections of human cross-facial nerve grafts demonstrated enhanced resolution of small-caliber thinly-myelinated regenerating motor axons. Resolution and contrast enhancement afforded by super-resolution imaging techniques enables visualization of unmyelinated axons, regenerating axons, cytoskeleton ultrastructure, and neuronal appendages of mammalian peripheral nerves using light microscopes.

## Introduction

Light microscopy is widely employed for histopathology. Conventional contrast and fluorescence-based light microscopy imaging techniques are diffraction limited to ~ 250 nm, precluding visualization of nanoscale structures. Though electron microscopy (EM) yields subcellular resolution, sample preparation is costly and time-consuming, and the technique is incompatible with living samples. Super-resolution microscopy (SRM) comprises fluorescence imaging techniques that circumvent the diffraction limit of conventional light microscopy to achieve superior resolution (Supplementary Fig. 1). SRM enables visualization of ultrastructural features of cells and tissues, rending it advantageous for biomedical research^1,2^.

Super-resolution microscopy comprises several techniques for resolution enhancement in optical microscopy. Patterned light illumination approaches include stimulated emission depletion (STED) microscopy^3,4^ and structured illumination microscopy (SIM)^5^. STED microscopy employs spatio-temporal overlap of a donut-shaped depletion beam with a Gaussian shaped excitation beam to restrict fluorescence emission to a region smaller than that limited by diffraction. SIM pairs scanning of an illumination pattern generated using a digital mirror with camera detection wherein camera pixels are employed as independent pinhole detectors for each spot in the pattern; acquired frames are post-processed using Fourier transforms to reconstruct a final image yielding enhanced resolution. Localization-based techniques include photoactivated localization microscopy^6^ and stochastic optical reconstruction microscopy^7^. Super-resolution radial fluctuations (SRRF) microscopy is an analytical approach that extracts super-resolution information from conventional fluorescence microscopy images by exploiting information encoded in the temporal fluctuation of fluorescence intensities across a sequence of images of a specific region-of-interest^8,9^. Recently, artificial intelligence algorithms have been employed for image optimization through denoising and resolution enhancement^10^.

Though EM remains the gold standard for nanoscale evaluation of nervous system tissue^11^, super-resolution light microscopy approaches are increasingly being employed. Super-resolution microscopy has expanded knowledge in the field of neurobiology through facilitating improved visualization of neural synapse structure and response to external stimuli. For example, use of stochastic optical reconstruction microscopy^12^ enabled the discovery that actin, spectrin, and associated proteins form a periodic structure in the cytoskeleton of axons, and elucidated the molecular architecture of synapses in the brain^13,14^. Rizzoli et al.^15^ employed STED microscopy to study the composition of synaptic boutons. STED microscopy has also been employed for live-cell imaging and morphologic quantification of dendritic spines^16^, and has permitted nanoscale visualization of neuronal appendages in the cerebral cortex of a living mouse^17^.

Heretofore, use of SRM in neuroscience has focused on investigation of the central nervous system. Though one recent study utilized STED microscopy to reveal the ultrastructural anatomy of the nodes of Ranvier within peripheral nerve^18^, SRM techniques remain underexploited in the field of peripheral and cranial nerve regeneration. Histomorphometric analysis is central to quantifying peripheral nerve repair outcomes in research settings yet is suboptimal when performed using images obtained with conventional light microscopy owing to suboptimal resolution of small caliber regenerating and unmyelinated axons. Herein, we employ several SRM techniques to reveal nanoscale features of rodent peripheral nerve and regenerating human cranial nerve samples, and compare results against images acquired by conventional widefield, confocal fluorescence, and transmission electron microscopy (EM).

## Methods

### Cell culture

Murine motor neurons (NSC-34) were fixed in 4% paraformaldehyde (PFA), stained for F-actin using AF488-phalloidin (A12379; ThermoFisher Scientific, Eugene, OR)^19^. Cells were plated on 18-mm #1.5 coverslips and mounted on glass slides (Superfrost Plus, Fisher Scientific, Pittsburgh, PA) using refractive index-matching media (Mount Liquid Antifade, Abberior GmbH, Göttingen, Germany).

### Animal tissue processing

All animal work was performed in accordance with Mass Eye and Ear Institutional Animal Care and Use Committee approved protocols, and all methods were performed in accordance with the relevant guidelines and regulations. Sciatic nerves from wild-type C57BL/6J mice were serially fixed in 2.5% glutaraldehyde and 2% osmium tetroxide solutions prior to resin embedding, ultramicrotome sectioning, and toluidine blue counterstaining for transmission electron microscopy (TEM) and light microscopy imaging as previously described^20^. Sciatic nerves were harvested from male and female adult Sox10-Venus (15–20 g) mice^21^. Sox10 is a transcription factor specifically expressed in oligodendrocytes and Schwann cells. Sox10-Venus mice express high levels of Venus fluorescence in Schwann cell nuclei and cytosol. Nerves from Sox10-Venus mice were fixed by immersion in 2% PFA, followed by overnight cryoprotection in sucrose solution, cryosectioning at 1 µm for widefield microscopy, and stained with a myelin-specific fluorescent dyes (FluoroMyelin Red, F34652 or FluoroMyelin Green, F34651 Invitrogen, Carlsbad, Calif.) as previously described^22^.

### Human tissue processing

Written informed consent was obtained from patients undergoing nerve transfer procedures at Mass Eye and Ear in accordance with Mass General Brigham Human Institutional Review Board approve protocols, and all methods were carried out in accordance with relevant guidelines and regulations. Biopsies of sural nerve autografts employed for cross-facial nerve grafting and housing axons of regenerating facial motor neurons were obtained fresh from patients undergoing second-stage free-muscle transplant for smile reanimation^23^. Regenerating nerve specimens measuring roughly 3 mm in length were immediately fixed by immersion in 2% PFA, followed by overnight cryoprotection in sucrose solution, cryosectioning at 2 μm, and staining with a myelin-specific dye (FluoroMyelin Green, F34651 Invitrogen, Carlsbad, CA).

### Brightfield/widefield microscopy

Samples were imaged using an upright microscope (Axio Imager A.2; Carl Zeiss, Oberkochen, Germany), with a 40× /1.3 and 100× /1.3 oil-immersion objective lens (EC Plan-Neofluar; Carl Zeiss), with transmitted light and reflected fluorescence observation using cooled charge-coupled device cameras (Axiocam 503 color and Axiocam 503 monochrome; Carl Zeiss). Cameras were equipped with sensors having a physical pixel length of 4.54 µm, yielding image pixel sizes of 113.5 nm/pixel (40× objective) or 45.4 nm/pixel (100× objective). Fluorescence was excited using a broadband light-emitting diode source (X-Cite 120 LED; Excelitas Technologies Corp., Waltham, MA), and a mercury lamp for brightfield illumination. Green fluorescence labelled samples were imaged using a GFP filter (Filter Set 09; Carl Zeiss) and red fluorescence labelled samples were imaged using a TxRed filter (Filter Set 00; Carl Zeiss). Images were acquired using a Zeiss software (ZEN 2Blue, Carl Zeiss, Carl Zeiss, https://www.zeiss.com).

### SRRF microscopy

Temporal stacks of 120 images were acquired with the widefield microscope to generate individual SRRF images. Sampling rates using 100X or 40X objective lenses were 45.4 nm or 144 nm, respectively. Images were acquired using the aforementioned mono CCD camera using a post-processing SRRF algorithm (NanoJ-SRRF^8^) in open-source image analysis software (ImageJ/Fiji^24^). The defaults parameters of SRRF algorithm such as ring radius (0.5), radiality magnification (5) and axes in ring (6) were used. Processing time of raw images (1936 × 1460 pixels) used to generate individual SRRF images was less than 10 min using a local performance PC (Intel^®^ Core™ i9-10920X, 128 GB DDR4 3200 MHz RAM).

### SIM microscopy

Widefield and SIM images were acquired using the ONI Nanoimager system (Oxford NanoImaging, Oxford, UK) using a 100× 1.41NA oil-immersion objective lens (PlanApo, Olympus, Tokyo, Japan). Excitation lasers at 488 nm and 561 nm were used for fluorescence excitation, with signal collected using respective FITC and TRITC filters via a dual-color channel sCMOS camera (ORCA-Flash4.0 V3, Hamamatsu Photonics) with an exposure time of 30 ms per frame. Image processing was carried out in parallel with frame acquisition.

### Electron microscopy

Sections were imaged using a transmission electron microscope (FEI Morgagni 268, Eindhoven, Netherlands) and images captured with a digital CCD camera (2K, Advanced Microscopy Techniques, Woburn, MA).

### Confocal/STED microscopy

Confocal and STED images were obtained on a commercial super-resolution microscope (Leica SP8X STED, Leica Instrument) using a high-aperture glycerol immersion objective lens (HC PL APO CS2 93× 1.30 GLYC, Leica Microsystems, Mannheim, Germany). Nerve sections from Sox10-Venus mice were excited using a pulsed 488 nm laser with a Gaussian beam profile and depleted by a donut-shaped 592-nm visible continuous wave laser. Fluorescence was collected with a hybrid detector using a spectral range of 500–550 nm and time-gated detection of 1 ns. Two dimensional confocal and STED images (2048 × 2048 pixels) were acquired with 2.5 times optical zoom, resulting in a frame size of 50 µm × 50 µm with 24 nm pixel size. The images were collected with a unidirectional scan speed of 1000 Hz, a pixel dwell time of 0.244 µs, and 16-frame averaging.

### Resolution estimation

Image resolution was assessed using the Fourier Ring Correlation (FRC) approach^25,26^, wherein images are analyzed in the frequency domain and resolution was estimated as inverse of the spatial cutoff frequency where the FRC curve drops below a value of 1/7. To estimate the global resolution of obtained images, a parameter-free approach was used to measure the degree of similarity of two independent images of the same region of interest. To estimate resolution achieved using SRRF microscopy; temporal image stacks (120 images) were separated into even and odd temporal frames to generate two new independent SRRF image stacks, which were then used to calculate the FRC. This method was run in Matlab using an available script^25^. A second method based on image decorrelation analysis^27^ was employed to estimate resolution from a single image achieved using widefield and SIM microscopy. The algorithm is parameter-free and is available as an open-source ImageJ plugin.

### SQUIRREL analysis

To further assess performance of the SRRF reconstruction, NanoJ-SQUIRREL^26^, a parameter-free analytical approach provided as an ImageJ plugin (Fiji Distribution, Version 1.52e, https://imagej.nih.gov) was employed. The plugin provides qualitative and quantitative assessment of super-resolution image quality and artifacts. Widefield and SRFF images collected on the same area were used as input for SQUIRREL analysis. An error-map was then calculated, wherein discrepancies between input and output images are identified, highlighting areas of the enhanced image that likely represent artifact. Overall image quality was assessed using the resolution-scaled Pearson coefficient (RSP), wherein the Pearson correlation coefficient between the diffraction-limited reference and resolution-scaled image is calculated; RSP values vary between -1 and 1.

### Image contrast

The image contrast (C) was calculated in MATLAB (MathWorks) as $\mathrm{C}=\frac{{\mathrm{I}}_{\mathrm{max}}- {\mathrm{I}}_{\mathrm{min}}}{{\mathrm{I}}_{\mathrm{max}}+ {\mathrm{I}}_{\mathrm{min}}}$. Where I_max_ and I _min_ are the maximum and minimum gray values of the image, respectively. Contrast enhancement (CE) between SIM and widefield image was calculated as $\mathrm{CE}(\mathrm{\%})=100\times \frac{{\mathrm{C}}_{\mathrm{SIM}}- {\mathrm{C}}_{\mathrm{WF}}}{{\mathrm{C}}_{\mathrm{WF}}}$.

### Image segmentation

Myelinated axons were segmented and quantified from digitized images of SRRF and widefield images using commercial machine learning software (Aivia v8.5, DRVision, https://www.aivia-software.com) as previously described^28^ using a local performance PC (Intel^®^ Core™ i9-10920X, 128 GB DDR4 3200 MHz RAM, NVIDIA Quadro RTX 5000). Briefly, a random forest pixel classifier was trained using regions of interest from individual images and thresholds applied to obtain final outputs (Supplementary Information and Supplementary Fig. 2). Relative error was calculated between automated myelinated axon counts and manual counting by three experts blinded to segmentation output and results compared between imaging techniques.

### Ethical approval

Human nerve samples herein were obtained from consenting patients undergoing nerve transfer procedures according to Mass Eye and Ear Internal Review Board approved protocols. Murine sciatic nerves were harvested after humane euthanasia in accordance with Massachusetts Eye and Ear Institutional Animal Care and Use Committee approved protocols. The study was carried out in compliance with the ARRIVE guidelines.

## Results

**Figure 1:**
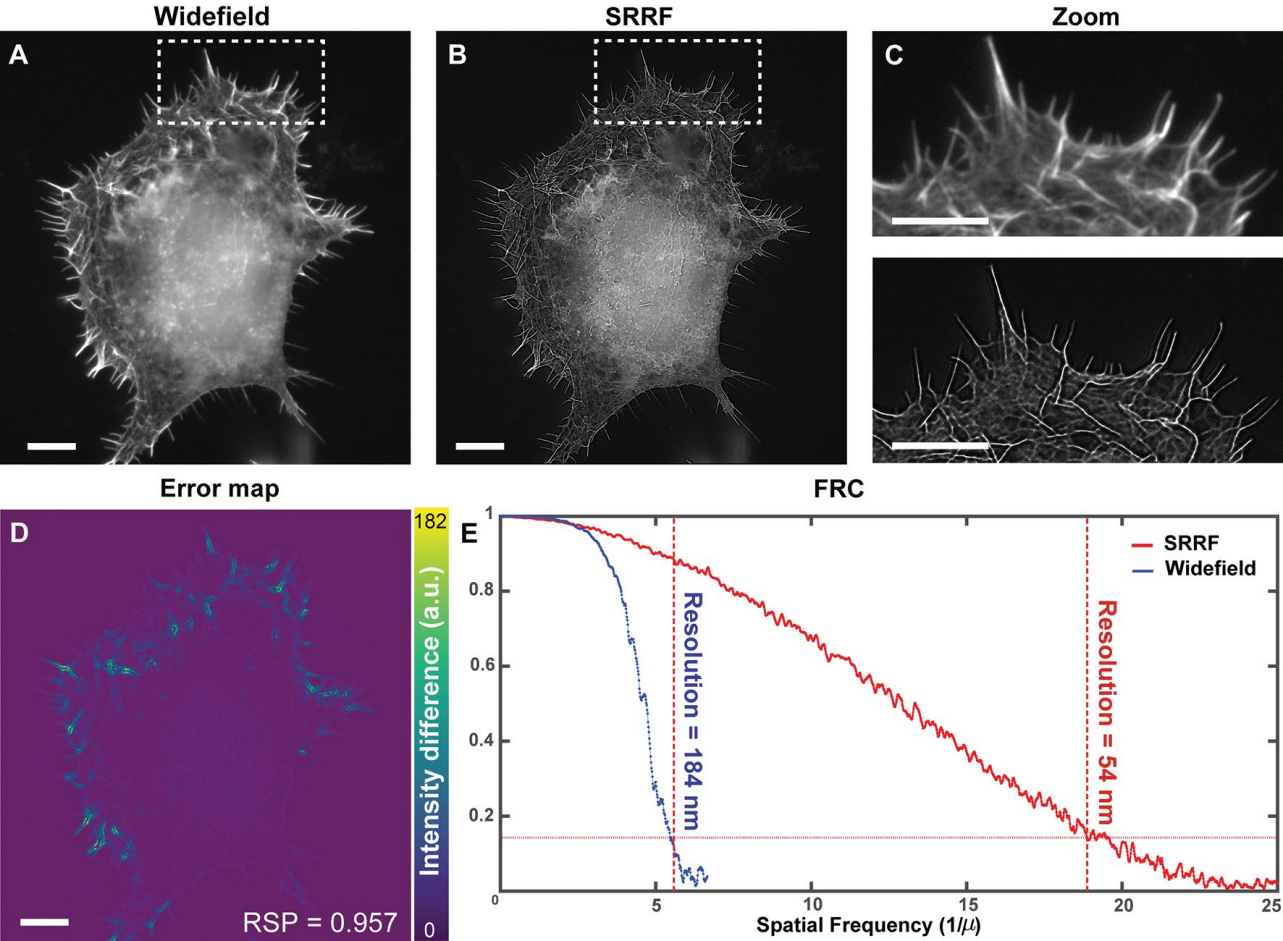
Super-resolution imaging of murine peripheral nervous system cells in culture. (**A–C**) The actin network labeled with Alexa 488 in cultured murine NSC-34 motor neurons as imaged with widefield and SRRF. (**D**) SQUIRREL analysis highlighting artifacts of SRRF reconstructions. (**E**) Resolution estimation using FRC curves for acquired widefield and SRRF images. Scale bar: 10 μm.

Images of murine-derived neuronal cultures using widefield and SRRF microscopy are shown (Fig. 1A–C). Super-resolution imaging demonstrated markedly enhanced resolution of the actin cytoskeletal network (Fig. 1D). SQUIRREL analysis indicated the SRRF images were reliable. The intensity and region of image artefacts are shown in the error map (Fig. 1D). The RSP value (0.957) near 1 indicated images were of high quality. The RSP average on similar images was 0.934 ± 0.033 (mean ± s.d.), see Supplementary Fig. 3. The image resolution for widefield and SRRF images was calculated using FRC curves (Fig. 1E). Resolution was enhanced from ~ 184 nm (σ = 13 nm, n = 3) to ~ 54 nm (σ = 5 nm, n = 3) representing a 3.4 fold improvement. Although images were acquired using 40× and 100× lenses with the same numerical aperture (1.3), greater resolution enhancement with SRRF microscopy was obtained for images acquired using the 100× lens owing to oversampling by a factor of 2.5 (45.4 nm pixel size).

**Figure 2:**
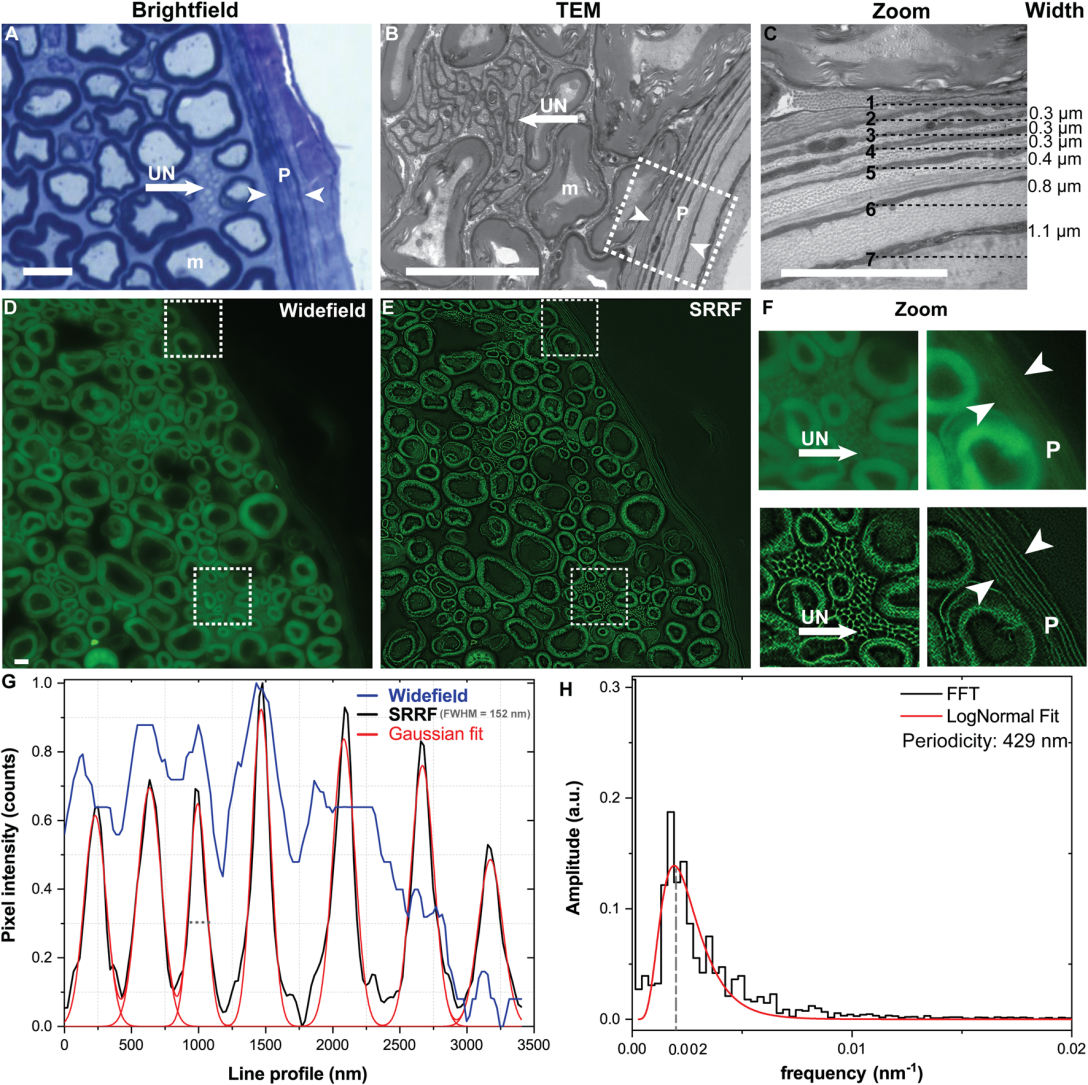
Imaging of murine peripheral nerve using light, electron and super-resolution microscopy. Wildtype murine sciatic nerve post-fixed with osmium tetroxide and counterstained with toluidine blue, and imaged using brightfield (**A**) and transmission electron (**B,C**) microscopy. Marked examples of myelinated fibers (m), unmyelinated fibers (UN, arrow), and perineurium (P, arrow heads) in the images. (**D–F**) Sox10-Venus sciatic nerve (expressing a GFP variant within Schwann cells) cross-section stained with FluoroMyelin Green and imaged using widefield fluorescence microscopy with resolution enhancement via post hoc SRRF processing. (**G**) Line profile along the direction of two arrowheads in (**F**) demonstrating resolution of seven individual layers of the perineurium using SRRF imaging, similar to that seen with EM in (**C**); layers cannot be resolved with widefield imaging (**D**). A Gaussian fitting was used to calculate the widths of the individual sheets. (**H**) Histogram of the fast Fourier transform analysis shows a periodicity of 429 nm in the cross sections of the perineurium. Gaussian fitting and fast Fourier transform analysis were done using Origin software (Origin 2021b, https://www.originlab.com). Scale bar 10 μm.

Brightfield imaging of sciatic nerve sections from wild-type mice yielded insufficient resolution to visualize unmyelinated axons, in contrast to the high resolution provided by EM (Fig. 2A–C). In contrast, SRM imaging of axial cryosections of sciatic nerve sections from Sox10-Venus mice demonstrated robust visualization of unmyelinated axons similar to EM imaging (Fig. 2D–F). To highlight concurrent visualization of myelin, Sox10-Venus sciatic nerve sections were labelled with a myelin-specific fluorescent dye. The perineurium—a connective tissue sheath encircling individual nerve fascicles—was visualized as a lamellar structure using EM and SRRF imaging (Fig. 2C,F). EM and SRRF imaging enabled resolution of individual lamellae of the perineurial sheath (Fig. 2C,F). Individual lamella thickness was calculated as full width at half maximum (FWHM) from multi-Gaussian fitted from line profile along the lamellar structures on SRRF images (Fig. 2G). A minimum image resolution of 152 nm was calculated from the FWHM of sharper lamella on the SRRF image. The calculated mean thickness of individual lamella was 190 ± 22 nm (n = 7). Mean inter-lamella distance measured from electron microscopy images (Fig. 2C) was 533 nm ± 309 nm (n = 6), compared to inter-lamella distances of 491 nm ± 91 nm (n = 7) measured from SRRF image as periodicity by Fourier analysis (Fig. 2H).

**Figure 3:**
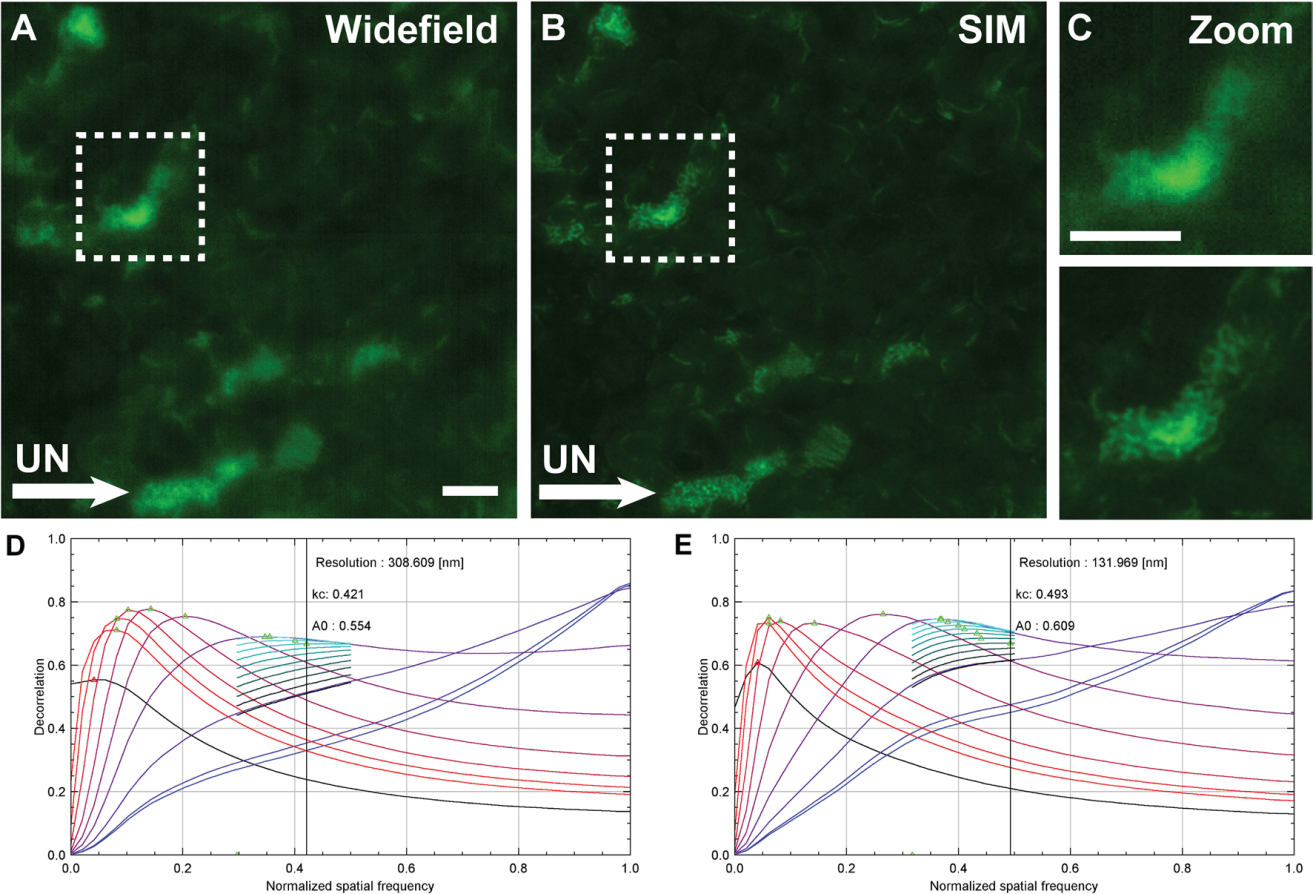
Super-resolution imaging of murine sciatic nerve cross-sections. (**A–C**) Fluorescent images of Sox10-Venus sciatic nerve imaged using widefield fluorescence and SIM microscopy demonstrates resolution enhancement of unmyelinated fibers. Marked examples of unmyelinated fibers (UN, arrow) in the images. (**D–E**) Resolution estimation using image decorrelation analysis. Scale bar 5 μm.

**Figure 4:**
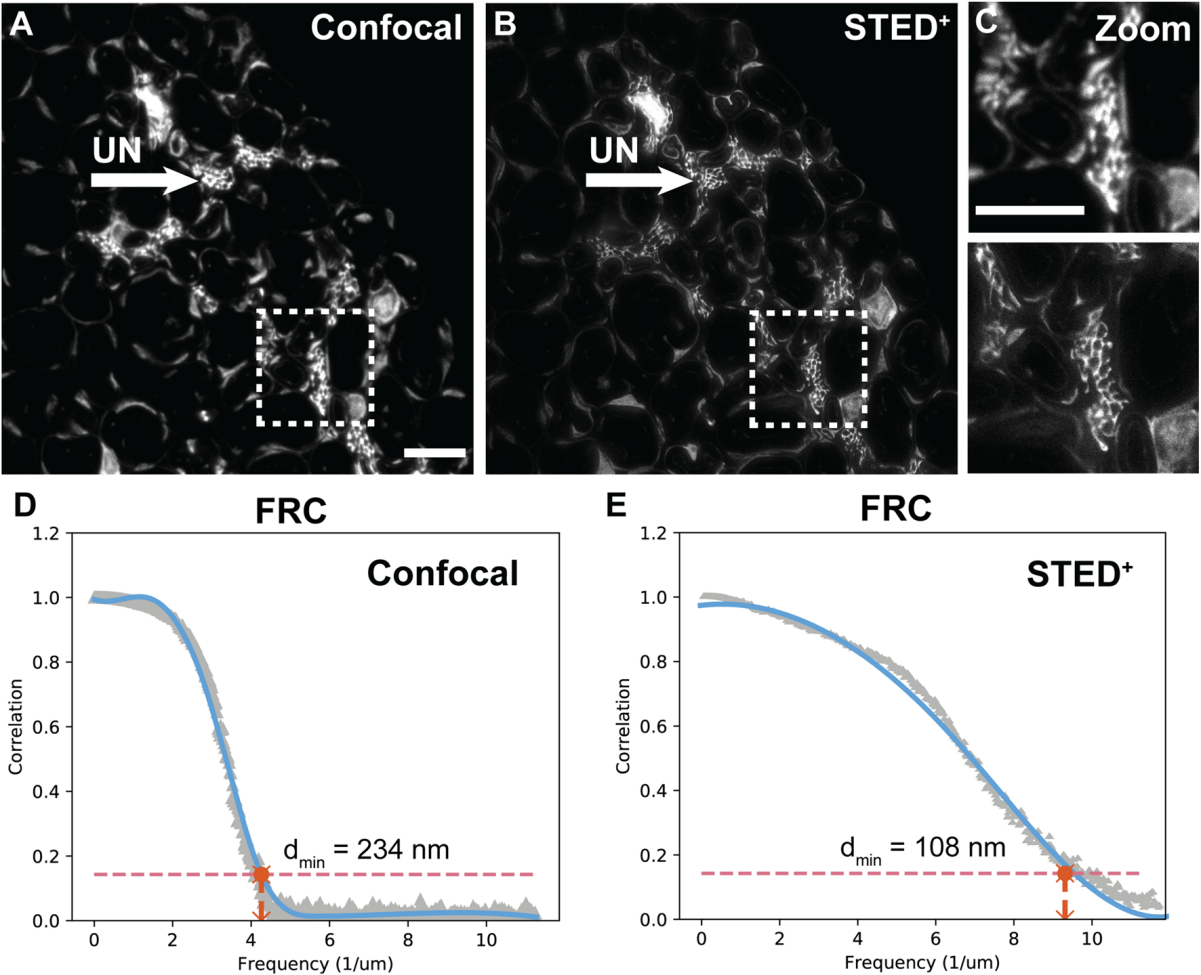
Super-resolution imaging of murine sciatic nerve cross-sections. (**A–C**) Fluorescent images of Sox10-Venus sciatic nerve imaged using confocal fluorescence and STED microscopy demonstrates resolution enhancement of unmyelinated fibers. Marked examples of unmyelinated fibers (UN, arrow) in the images. Resolution estimation using FRC. Scale bar 5 μm.

SIM imaging enabled enhanced visualization of unmyelinated axons in Sox10-Venus sciatic nerve, without blurring typical of widefield fluorescence imaging (Fig. 3A–C). A contrast enhancement of 21.5% was observed in SIM images compared to widefield images. Image decorrelation analysis was employed to estimate resolution; compared with widefield, SIM microscopy yielded a 2.3-fold enhancement in resolution (Fig. 3D–E). Use of STED microscopy also yielded improved resolution of unmyelinated axons in Sox10-Venus sciatic nerve (Fig. 4A–C). The resolution of STED images was further improved using a deconvolution technique (Fig. 4D–E) proposed by Koho et al.^29^, which employs FRC to estimate the effective point-spread-function directly from the STED images.

**Figure 5:**
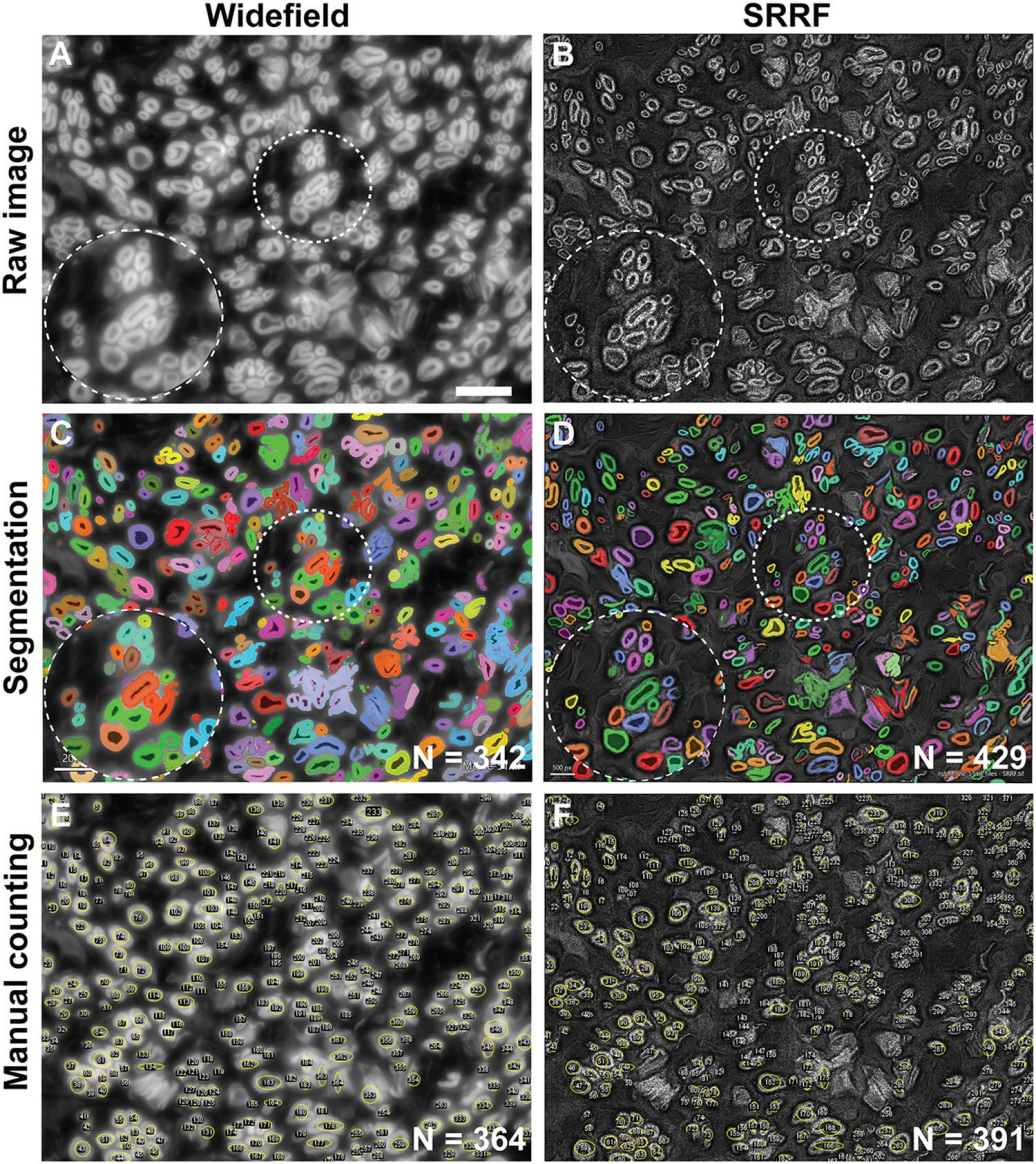
Super-resolution imaging of regenerating human nerve labelled with a myelin-specific dye. Biopsy of a human cross-facial nerve autograft harvested at time of functional muscle transfer, cryosectioned at 2 µm, and stained with FluoroMyelin Green. (**A,B**) Resolution enhancement using SRRF imaging is demonstrated in comparison to widefield fluorescence imaging. (**C,D**) Resulting image segmentation using machine learning based software. Inset: magnified view of circled areas. (**E,F**) Manual counting of widefield and SRRF images performed by a blinded expert. Pixel sizes: 45.4 nm; Field of view: 88 μm; Scale bar 10 μm.

SRRF imaging of regenerating human nerves demonstrated enhancement of effective resolution (Fig. 5A,B). The inset of Fig. 5 (zoom) compares imaging and subsequent segmentation of regenerating axons using widefield fluorescent microscopy and SRRF. The mean myelin-sheath thickness of regenerating fibers was 260 nm ± 78 nm (n = 34), calculated using a line profile on SRRF images in ImageJ software. Owing to improved resolution, axon segmentation results from SRRF microscopy images demonstrated higher accuracy while requiring fewer training rounds to obtain reliable segmentation in comparison to widefield images (see Fig. 5C,D). Owing to the higher pixel count typical of SRM images, computation time for pixel classifier training and inference was prolonged for SRRF images compared to widefield images. Pixel classifier training times for widefield and SRRF images was 2.38 s and 12.42 s, respectively. Inference times for widefield and SRRF images were 504 ms and 2.18 s, respectively. The thickness of myelin rings of regenerating nerve fibers ranged between 150 and 1000 nm. The relative error between automated and manual axon counts for widefield and SRRF images was 12% and 9%, respectively. The resolution enhancement afforded by SRRF microscopy enabled improved segmentation of abutting axons and hence more accurate histomorphometric analysis.

## Discussion

This report highlights the potential of SRM to improve qualitative and quantitative assessment of the peripheral nervous system. Resolving nanoscale structures in histopathologic samples using light microscopy is challenging owing to the complexity, heterogeneity, and thickness of biologic tissue. This is the first report on the use of SRM to evaluate regenerating human peripheral nerve. Thinly myelinated regenerating nerve fibers poorly resolved with widefield microscopy were readily visualized using SRM techniques. Notably, such fibers were labelled using a rapid and nontoxic fluorescent dye readily applied to frozen or fixed nerve sections^22^. These myelin-specific dyes were found to be compatible with SRRF and SIM techniques. Further, these dyes were found to adequately stain the perineurium, allowing resolution of its lamellar structure^30^. Future work might explore the use of exchangeable labels, which may enable STED microscopy imaging of membrane structures^31^. Prior quantification of regenerating human facial nerve axons in cross-facial nerve grafts has necessitated use of EM^32^. Herein, the potential of SRM light microscopy techniques and AI-based segmentation to quantify the number of regenerating axons within human nerve sections using an efficient frozen section approach was demonstrated. The accuracy of automated axon quantification obtained with commercial software was demonstrated by direct comparison with manual axon counting. Such capability carries potential to inform intra-operative surgical decision-making in peripheral nerve transfer and repair procedures by enabling rapid confirmation and quantification of the extent of axonal regeneration within a donor nerve^22,33^.

Traditional neural histomorphometry is focused on quantification of myelinated axon counts and their morphology. As visualization and quantification of unmyelinated fibers has heretofore required labor-intensive electron microscopy, unmyelinated fibers are frequently excluded in histomorphometric assessment of peripheral nerve^33^. Visualization of small unmyelinated fibers is limited in conventional widefield imaging due to poor resolution and out-of-focus fluorescence light. Using SIM and STED microscopy, enhanced image contrast and resolution enabling visualization of unmyelinated fibers was observed herein (Figs. 2F, 3C, Supplementary Fig. 4C). Unmyelinated fibers were also resolved with cost-effective SRRF microscopy. Quantification of the number and size of unmyelinated and thinly-myelinated axons is advantageous in the study of nerve regeneration following injury^27,33^; improved means to resolve unmyelinated and thinly-myelinated fibers may also help advance understanding of axon regeneration, sensory neuropathies, and pain disorders. Beyond its use for peripheral nerve histomorphometry, SRM carries potential to inform clinical diagnosis and management of other disease states including glomerulonephritis, viral infections, and metabolic diseases such as amyloidosis currently based on labor-intensive electron microscopy techniques for diagnosis.

Table 1 provides a comparison of SRM techniques employed herein. Purely optical versus computational SRM approaches have opposing advantages and disadvantages. Though STED microscopy may be performed on samples up to 100 µm thick, SIM and SRRF techniques are based on widefield approaches that require thin (< 10 µm) sections of low-scattering biological samples to minimize out-of-focus fluorescence signal that would otherwise degrade image reconstruction. Though SRM imaging of cell components may achieve lateral resolutions on the order of tens of nanometers, highly scattering histologic tissue lowers the effective resolution of these techniques in most instances to ~ 100 nm. Use of optically-based SRM techniques including STED are limited by complexity and cost, whereas computational approaches are cost-effective. The analytical-based SRRF approach is a cost-effective solution based on open-source software^24^ to upgrade a conventional widefield microscope for SRM capability.

**Table 1:** Comparison of super-resolution imaging techniques.

Application	SRM technique		
	SRRF	SIM	STED
Lateral resolution	50–150 nm	110–150 nm	30–110 nm
Illumination	Low excitation intensities (mW/cm^2)^	Medium excitation intensities (W/cm^2^)	High excitation intensities (kW/cm^2^)
Post-processing	Radiality transform & temporal correlations	Fourier transform	Optional (deconvolution)
Complexity	Low	Medium	High
Multicolor	4-color	4-color	2-color
Sample thickness	< 10 µm	< 10 µm	< 100 µm
Cost	Highly cost-effective	Moderate cost	Costly

In contrast to computational SRM approaches such as SRRF and SIM, purely optical SRM techniques such as STED avoid the risk of image artefacts. Common artifacts within SRRF images include shadowing at structural boundaries and star-shaped patterns in circular structures^34^. Image quality (resolution and artifacts) in SRRF is degraded by suboptimal imaging conditions including low acquisition frame rate or sub-Nyquist sampling, and suboptimal selection of SRRF algorithm parameters including inappropriate ring radius or radiality magnification. NanoJ-SQUIRREL is a robust imaging tool to localize artifacts on super-resolved images and to guide optimal parameter settings for image acquisition using SRRF microscopy^26^.

STED microscopy requires specific bright and photostable fluorescent dyes, whereas SIM and SRRF microscopy may be achieved using nearly any fluorescent dye. In STED microscopy, two-to-eight-fold improvement over confocal resolution is obtained by increasing the power of the depletion beam in STED microscopy, with achievable resolution determined by the intensity of the depletion beam that can be focused on the sample without photobleaching. Computational approaches typically require far less excitation powers, rendering them more compatible with live-cell experiments in comparison to STED approaches. STED microscopy is generally limited to super-resolved imaging of two colors, typically implemented using 595 nm and 775 nm depletion lasers, whereas purely computational approaches are limited only by the number of dyes and corresponding appropriate filter cubes available. As STED microscopy is a purely optical technique, the use of post-processing image deconvolution is optional to further increase effective resolution. Herein, deconvolution of STED images was employed to recover frequencies beyond the diffraction limit (Supplementary Fig. 5).

Herein, resolution of thinly-myelinated regenerating axons in human nerve grafts was demonstrated using frozen-section techniques. Segmentation of small caliber regenerating axons having diameters near the diffraction limit of light is challenging using conventional light microscopy. Figure 5 demonstrates clear resolution enhancement and improved segmentation of regenerating axons where SRM techniques were employed. We anticipate SRM will become indispensable in nerve histology for visualization and quantification of thinly-myelinated regenerating fibers and other subcellular structures below the diffraction limit of visible light. Future work will systematically compare histomorphometric assessment of regenerating peripheral nerve using rapid SRM fluorescent-labelling techniques versus conventional resin-embedded EM imaging approaches. Jacobs et al. demonstrated a majority of regenerating fibres in human cross-facial nerve grafts are unmyelinated, suggesting these axons carry potential to become myelinated following target neurotization^33^. Future work will seek to resolve promyelinated and unmyelinated axons on rapid frozen sections by means of SRM techniques paired with adaptive illumination of fluorescent-labelled axonal membranes. Though resource-intensive resin-embedding was previously thought necessary for preservation of the structural integrity of nanoscale unmyelinated fibers during sectioning^35^, work by our group herein and elsewhere^36^ has demonstrated the suitability of cryosection approaches for resolution of unmyelinated fibers using light microscopy.

## Supplementary Information

Supplementary Information.

## Data Availability

The data that support the findings of this study are available from the corresponding author upon reasonable request.
