# Supplementary material for: Super-resolved fluorescence imaging of peripheral nerve
Source: Sci Rep. 2022 Jul 21;12:12450. doi: 10.1038/s41598-022-16769-0 (PMC9304405; doi:10.1038/s41598-022-16769-0)
Supplement: Supplementary file 1 — Supplementary Information. [file 41598_2022_16769_MOESM1_ESM.docx]

**Supplementary Information**

**Super-Resolved Fluorescence Imaging of Peripheral Nerve**

Iván Coto Hernández^1,*^, Suresh Mohan^1^, Steven Minderler^1^ & Nate Jowett^1,*^

^1^Surgical Photonics and Engineering Laboratory, Mass Eye and Ear, Harvard Medical School, Boston, Massachusetts

^*^ Corresponding author: [ivan_cotohernandez@meei.harvard.edu](mailto:ivan_cotohernandez@meei.harvard.edu);

[nate_jowett@meei.harvard.edu](mailto:nate_jowett@meei.harvard.edu)


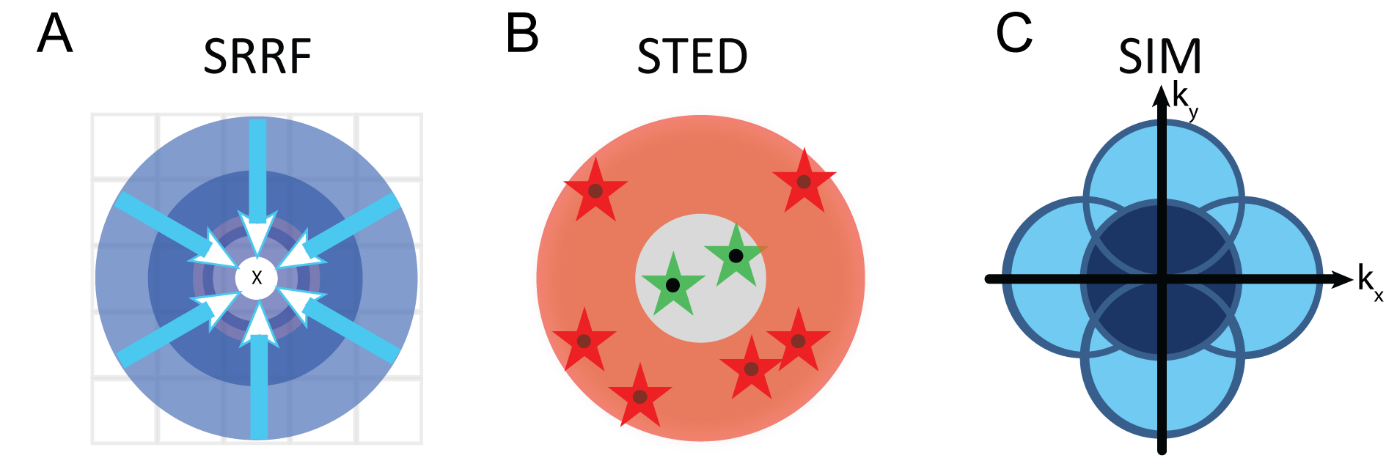


**Suppl. Fig. 1. Basic principles of various super-resolution fluorescence microscopy techniques.** (A) The SRRF algorithm calculates the intensity gradient convergence for each frame on sub-pixel regions. The figure shows a two-dimensional plot of the gradient magnitude, where the arrows indicate the direction of local intensity gradient. The degree of convergence of these vectors at the central subpixel (x) is used for further temporal correlation analysis of an image sequence to form the final SRRF image. (B) The STED laser is focused into a doughnut-like pattern (orange) and aligned with the diffraction-limited focal spot of an excitation laser to confine the detection volume to sub-diffraction dimensions (gray). (C) Schematic representation of spatial frequencies observable by linear structured illumination with a two-dimensional pattern. The dark blue circle indicates the observable region of spatial frequencies in a conventional microscope. Scanning the structured illumination increases by a factor of 2 the lateral resolution and optical sectioning.

**Image segmentation**

A commercial machine learning software (Aivia v8.5, DRVision Technologies LLC, Bellevue, WA) was used for segmentation of myelinated axons. The segmentation algorithm uses a machine-learning based pixel classifier and thresholding in individual images. A trainable pixel classifier is based on a random forest algorithm, a traditional machine learning approach using decision trees whose results are combined into a final result. The Suppl. Fig. 2A, B shows an example of the small datasets needed for training (green highlights the desired segmentation region, while red highlights the background). The segmentation algorithm uses additional thresholding parameters - detection, partitioning, and size - to fine tune the final segmentation output. These parameters are dynamically adjusted in a user-friendly way by moving a slider control from left to right to cover the total range of the image.

• The detection parameter specifies the confidence threshold for the segmented objects. Reducing the minimum confidence threshold results in detection of more low-confidence objects as well as larger and more connected objects.

• The partition parameter specifies the aggressiveness of the partitioning algorithm applied to the segmented regions. Increasing the aggressiveness of partitioning results in more partitioned objects.

• The object size parameter specifies the object size range based on the user-drawn regions. The lower threshold modifies the minimum size and the upper threshold modifies the maximum size.


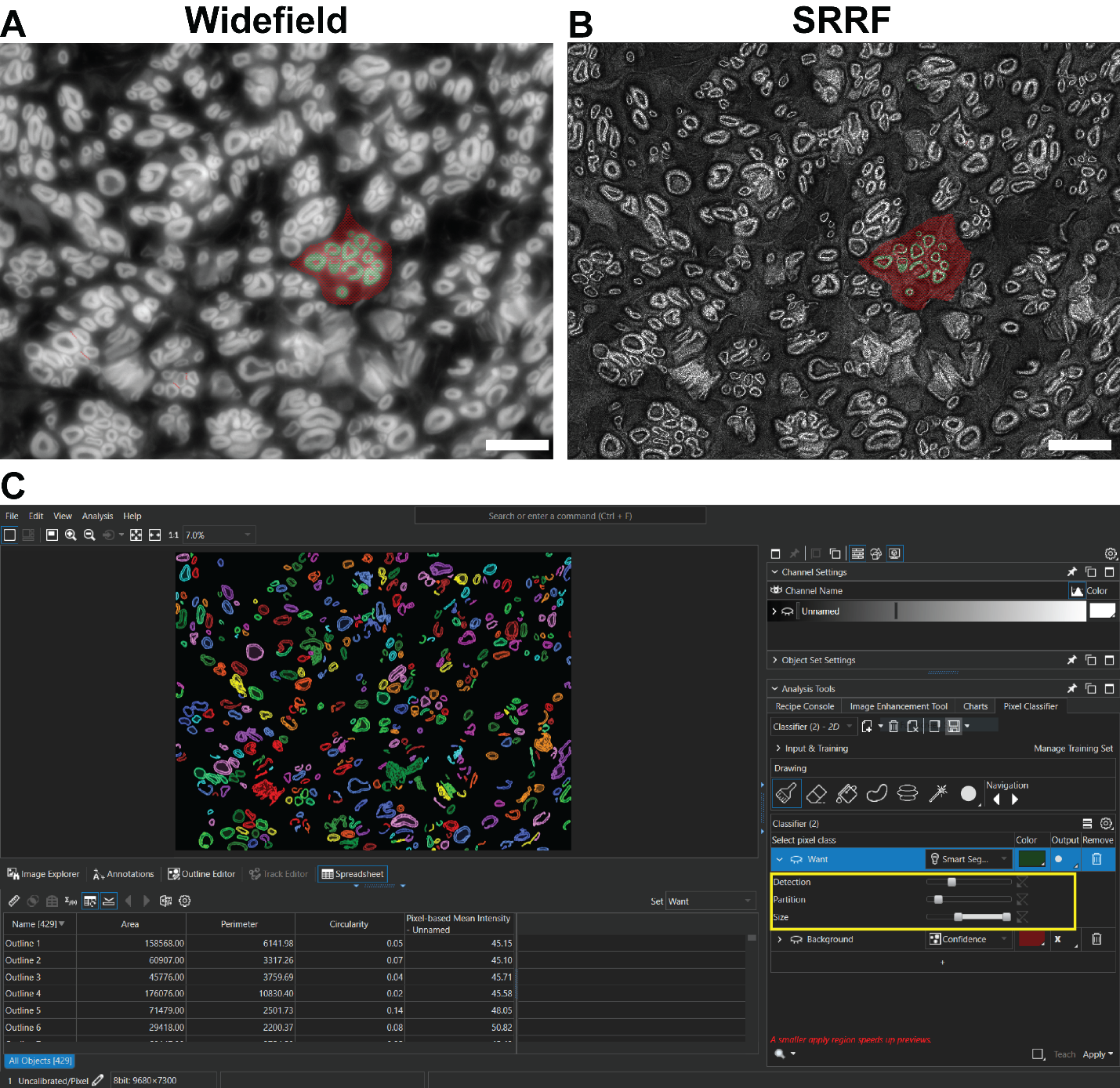


Suppl. Fig. 2. Image segmentation of regenerating human nerve labelled with a myelin-specific dye. (A-B) Comparison between widefield and SIM images. The inset shows labeled examples of training regions employed for segmentation of widefield and SRRF images, respectively. (C) Screenshot of the machine learning software employed for counting myelinated axons. Scale bar 10 μm.

**
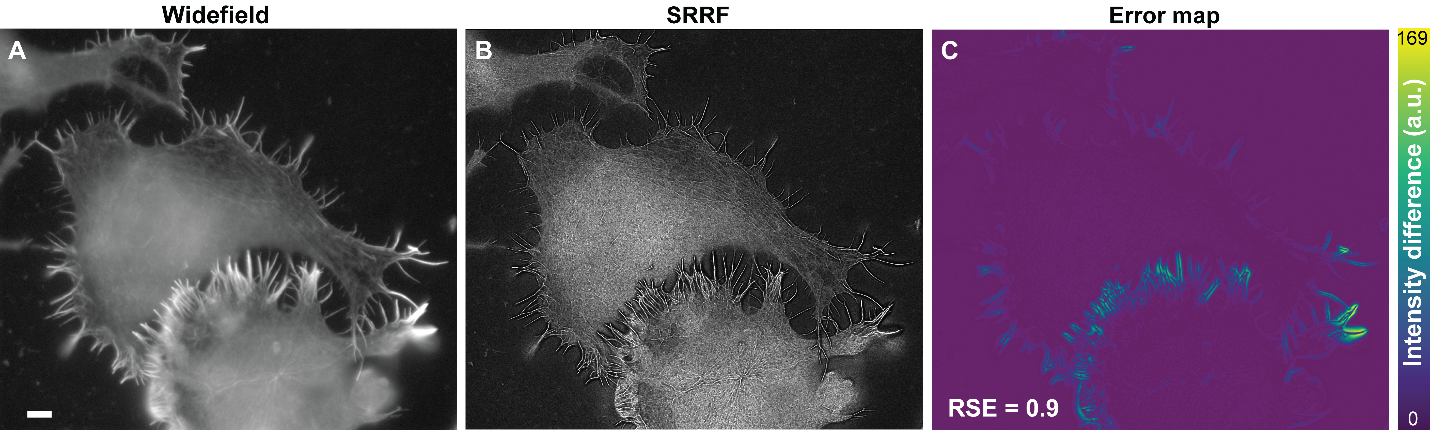
**

**Suppl. Fig. 3. Super-resolution imaging of murine peripheral nervous system cells in culture.** (A-B) The actin network labeled with Alexa 488 in cultured murine NSC-34 motor neurons as imaged with widefield and SRRF. (C) SQUIRREL analysis highlighting artifacts of SRRF reconstructions. Scale bar 10 μm.

**
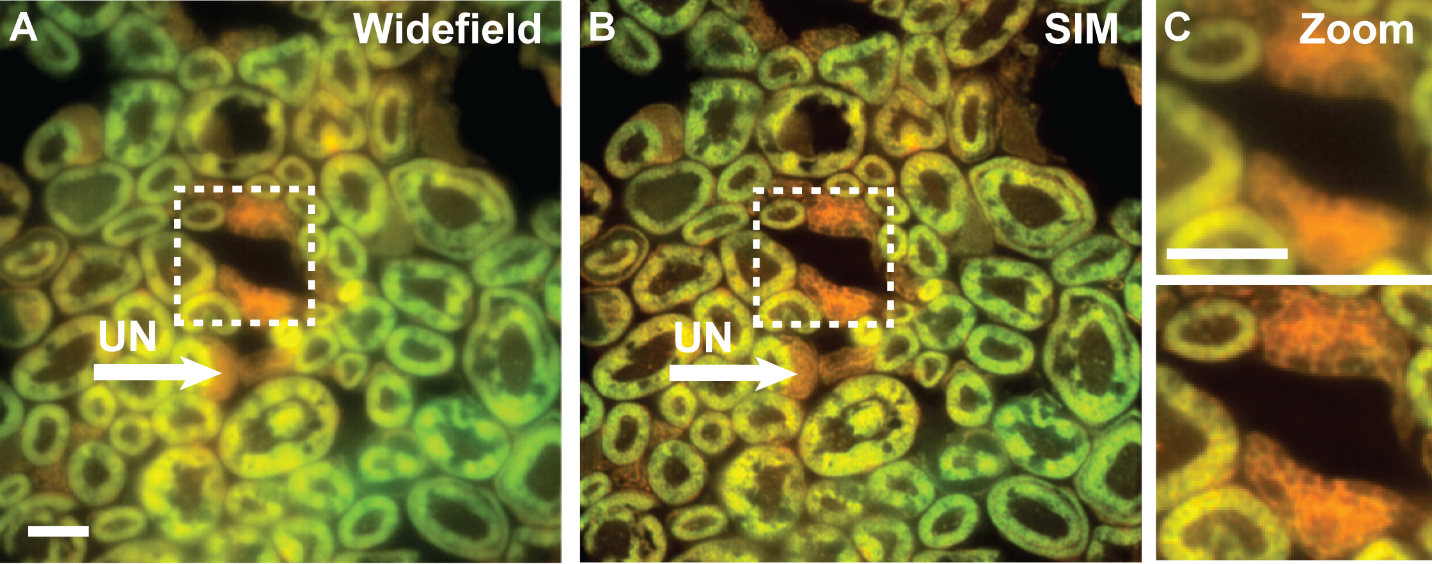
**

**Suppl. Fig. 4. Super-resolution imaging of murine sciatic nerve cross-sections.** (A-B) Composite image of Sox10-Venus sciatic nerve stained with FluoroMyelin Red and imaged using widefield fluorescence and SIM microscopy demonstrates resolution enhancement of unmyelinated fibers. Marked examples of unmyelinated fibers (UN, arrow) in the images. Scale bar 5 μm.


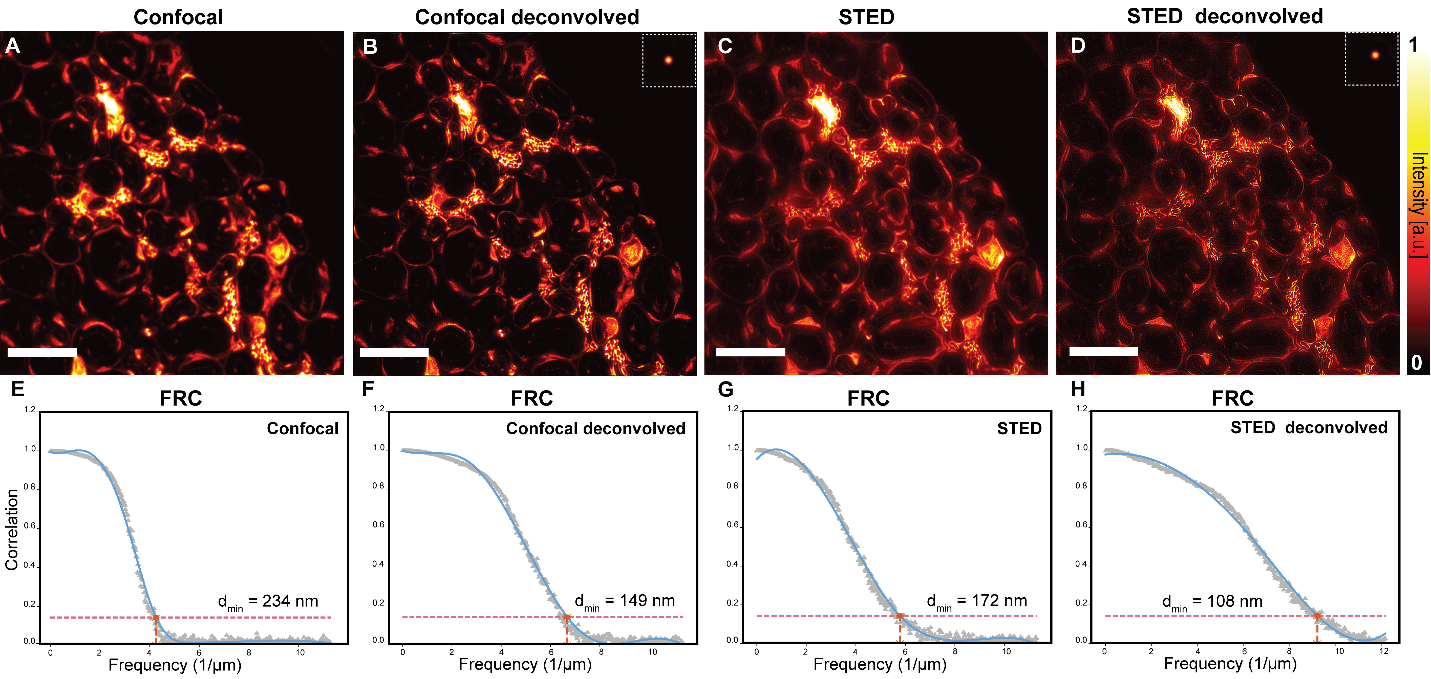


**Suppl. Fig. 5. Comparison between confocal and STED imaging of sciatic nerves of Sox10-Venus mice.** (A-C) Comparison between confocal, confocal deconvolved, STED and STED deconvolved images. Deconvolved images were obtained after 50 iterations. The inset of figures B and D show a zoom of the point spread function used for deconvolution. Note panels from Fig. 4 of the manuscript (Panels A,B,D,E) are also selections of this panel. (E-H) Resolution assessment between confocal, confocal deconvolved, STED and STED deconvolved using the FRC curves. Scale bars: 10 µm.
